# Effectiveness of a self-management support program for type 2 diabetes patients in the first years of illness: Results from a randomized controlled trial

**DOI:** 10.1371/journal.pone.0218242

**Published:** 2019-06-27

**Authors:** Anne L. van Puffelen, Mieke Rijken, Monique J. W. M. Heijmans, Giel Nijpels, François G. Schellevis, on behalf of the Diacourse study group

**Affiliations:** 1 NIVEL (Netherlands Institute for Health Services Research), Utrecht, the Netherlands; 2 Department of General Practice & Elderly Care Medicine, Amsterdam Public Health Research Institute, Amsterdam University Medical Centers, location VUmc, Amsterdam, the Netherlands; Florida International University Herbert Wertheim College of Medicine, UNITED STATES

## Abstract

**Aims:**

To evaluate the immediate and six-month effectiveness of a group-based self-management support program for people diagnosed with type 2 diabetes (1–3 years post diagnosis) on diabetes self-care, distress and cognitions.

**Methods:**

People with type 2 diabetes were randomized into the intervention (four group-based interactive sessions) or the control group (a single educational lecture) with their partners. Outcomes were measured at baseline, immediately after the third course session and six months later using validated questionnaires on diabetes self-care, distress, illness perceptions, diabetes-related attitudes, empowerment and partner support. Multilevel analyses were conducted according to the intention-to-treat principle using the data from 82 intervention and 86 control group participants, to test for differences in changes over time between the two groups.

**Results:**

The intervention group showed a significantly higher increase in physical activity and fruit and vegetable intake immediately after the program, whereas the low baseline levels of diabetes distress remained unaffected. Furthermore, the intervention group believed their illness to be more likely to be caused by chance/bad luck, but also felt more empowered to handle their condition and its treatment immediately after the program compared with the control group. Six months later, only the differences in empowerment had persisted.

**Conclusions:**

Group-based self-management support results in favorable short-term behavioral changes and more persistent alterations in (perceived) empowerment in people living in the first years of type 2 diabetes. In order to achieve more sustainable behavioral changes, more prolonged support is necessary. This could be achieved by integrating attention to patients’ illness perceptions and continuous self-management support in regular diabetes care.

**Trial registration:**

Netherlands Trial Registry NL3158.

## Introduction

People with type 2 diabetes (T2DM) face the challenge of adapting to living with a chronic and progressive condition on a daily basis. From diagnosis, people with type 2 diabetes need to adopt a multifaceted behavioral treatment regimen, to diminish the risk of developing and/or deteriorating diabetes-related complications [1] and, consequently, to maintain adequate functioning and a satisfactory quality of life [2–4]. However, the day-to-day management of chronic illness is often considered challenging, and half of the people with chronic conditions in the Netherlands appears to struggle with making the recommended behavioral changes [5]. Moreover, in the absence of symptoms, people with type 2 diabetes generally underestimate the (potential) seriousness of their condition and, consequently, the importance of engaging in self-care [6]. Support focusing on successful strategies to incorporate diabetes self-management within daily life might therefore be particularly warranted during the first years after diagnosis.

Previous studies on diabetes self-management support have emphasized the importance of focusing on illness perceptions and one’s social environment, in addition to self-efficacy, as important determinants for health behaviors and outcomes [7–9]. With this in mind, we developed a self-management support program based on the Common-Sense Model of self-regulation (CSM) [10,11], while also incorporating principles from Social Cognitive Theory (SCT) [12,13] and social support theories [14–16]. According to the CSM, individuals make sense out of their condition by forming personal models about the illness and its treatment, which determine their coping responses and, consequently, influence health-related outcomes. Such personal models comprise several cognitive and emotional dimensions, with ‘perceived seriousness’ and ‘perceived control’ being the most influential on behavioral and emotional management [7,17]. In a rather similar way, the SCT states that behavior change will only be elicited when the outcomes are deemed beneficial and important by the individual (outcome expectancies), and (s)he is confident in his or her ability to perform these behaviors (self-efficacy). Providing medical and practical information, sharing experiences in diabetes management, and teaching individuals how to set realistic and manageable goals and develop action plans are therefore considered effective strategies in diabetes self-management support [18]. Furthermore, as social support theories state that support from partners (or other close friends and relatives) can both activate and inactivate patients’ behavioral and emotional management [19–21], it is of great importance that patients’ “important others” are involved in self-management support programs. Hence, by addressing patients’ and partners’ illness perceptions, setting realistic goals and developing concrete action plans, and by stimulating activating support from partners or significant others, we aimed to improve type 2 patients’ self-management and quality of life in the first years of living with the disease.

So far, comparable support programs have mainly targeted participants’ self-efficacy in their efforts to improve self-management in T2DM; a strategy that appears to yield positive, yet primarily modest and short-term results [22–25]. Considering the important role of illness and treatment perceptions in patients’ willingness and (perceived) ability to engage in health behaviors [6,7,17], we believe that these personal beliefs should be the main focus and starting point of our intervention. In addition, tailoring the intervention to the specific needs and challenges that are inherent to the different phases in living with diabetes might increase and prolong its (potential) effects. Two studies previously tested the effectiveness of CSM-based support programs in type 2 diabetes patients, one directly after diagnosis [26,27] and the other in patients with poor glycemic control [9]. The program we developed specifically targeted patients who had been diagnosed one to three years ago; a phase in the illness process where the initial attention paid to patient education and support often fades away—especially in the absence of diabetes-related problems or complications–and where many patients have already been confronted with barriers that impede making and maintaining behavioral changes [6,28]. Self-management is a broad concept, comprising cognitive, behavioral and emotional aspects of living with a (chronic) condition [29,30]. For the purpose of this study, we chose to focus on the behavioral component of self-management, being the self-care behaviors and lifestyle recommendations that are part of the diabetes treatment regimen. In addition, our study focused on diabetes distress being an important diabetes-related aspect of health-related quality of life.

The present article reports the effectiveness of this support program on patients’ diabetes self-care and distress as primary outcomes, both immediately after the intervention and at six months follow-up. We also assessed the immediate and follow-up effects of the intervention on more proximal secondary outcomes, including diabetes-related perceptions and attitudes, empowerment and perceived partner support, which enabled us to gain more insight in the presumed working mechanisms of the intervention. We hypothesized that the group-based self-management support program would result in participants: (a) performing more (appropriate) diabetes self-care (including healthy behaviors/lifestyle aspects) and (b) experiencing lower levels of diabetes-related distress immediately after the program and six months later, as compared to a control group. Furthermore, we hypothesized that the group-based self-management support program would result in participants: (a) holding more adaptive illness perceptions and attitudes towards their condition; (b) feeling more empowered to manage their condition, and c) experiencing more activating partner support, immediately after the program and six months later, as compared to a control group. Although it cannot be stated that certain illness beliefs are always adaptive (or maladaptive), perceiving diabetes to be a serious condition, while also perceiving its course and consequences to be (to a certain extent) controllable by a healthy lifestyle, appropriate self-care and medical treatment is generally considered an adaptive personal model of T2DM. A sense of understanding the illness may also contribute to adaptive health and illness behaviors [7,17]. Similarly, active engagement of partners in helping patients use constructive problem solving skills is generally found to be adaptive, whereas protective buffering and overprotection by partners may negatively impact their (psychological) health and illness behaviors [20,31,32].

## Materials and methods

A randomized controlled trial (RCT) was carried out, comparing the effects in an intervention group to a control group. Outcome measures were assessed at baseline, immediately after the intervention program (two months from baseline) and at six months follow-up (eight months from baseline). Written informed consent was obtained from participating patients prior to the start of the study. The study was approved by the Medical Ethical Committee of the VU University Medical Center Amsterdam (VUmc) and registered in the Dutch trial register [NL3158]. The protocol of the study has been published previously [33].

### Participants

People with type 2 diabetes and an illness duration between one and three years post-diagnosis who received diabetes care from general practices (GPs) in six regions of the Netherlands were included in the study between the summer of 2012 and the summer of 2013. Almost all GPs in the Netherlands are organized in diabetes care groups, which may consist of 50 to more than 200 GPs who together agree upon a diabetes management program that is contracted by health insurers. For this study, we approached nine diabetes care groups of which six eventually participated and distributed information about the study and a request for participation to all their affiliated GPs. GPs willing to participate signed up for the study directly with the researchers. Subsequently, patients registered with these GPs, who had been diagnosed between one and three years ago, were selected. First, an electronic selection of all T2DM patients in the participating GP’s was performed, based on ICPC code (T90.02). From there, all further data were selected by manual abstraction of the electronic medical records. People were excluded if they were 1) over the age of 85, 2) unable to speak, read and/or understand the Dutch language sufficiently, 3) mentally or intellectually incapable to participate, or 4) suffering from a severe life-threatening condition (e.g., specific types of cancer) or currently receiving (psycho)therapy for severe psychological or psychiatric problems, as reported by their GP or practice nurse.

Eligible people received a written invitation for participation, a generic participation recommendation letter from their GP, and an answer form to indicate on whether or not they agreed to participate. Invited people were also asked to complete a three-item screening questionnaire, assessing diabetes-related uncertainty, coping with diabetes and perceived consequences of diabetes (see S1 Table). Patients with a total sum score of 0, indicating that they did not experience any uncertainty or problems in coping with their illness nor perceived any negative consequences of diabetes at that moment were considered as non-eligible, as they were not expected to benefit from the program. Hence, these people were excluded from the study before randomization.

### Intervention and control condition

Randomization to the intervention and control condition was electronically performed at patient level, stratified per region, by a researcher who was not involved in the study. All participants were actively encouraged to bring their partner or, when a partner was absent, a close friend or relative to the course sessions (intervention) or information meeting (control condition).

#### Intervention

Participants in the intervention group were invited to a group-based self-management support program, consisting of three monthly 2-hour interactive sessions and one booster session three months after the last session. All sessions were led by two diabetes nurses or practice nurses who received a four-hour training, and a detailed manual describing the content of the program and its underlying theories to be used during the sessions. Participating patients and their partners received a workbook which contained basic information about diabetes, (homework) assignments, and theoretical and practical information about the topics discussed during the course (both books are available in Dutch from the corresponding author).

The content and method of delivery of the course were first derived from literature study and the results from a focus group. Subsequently, the manual for the (future) course leaders and the work book for the participants were developed and pilot tested in two groups. The experiences with the intervention during the pilot were used to adapt the content and method of delivery of the sessions, and served as input for the training of the future course leaders. Finally, the researcher and health psychologist that observed and guided the pilot trained the diabetes nurses and practice nurses to deliver the intervention during the RCT. The content of the three course sessions and the booster session is outlined in Table 1.

**Table 1:** Outline of the living with diabetes course.

	Aim	Activity	Main related outcome
**Session 1**			Perceptions/attitudes
Discussing illness perceptions.	Creating awareness on differences in perceptions and their link with behaviors.	Discussing the different perceptions on seriousness, controllability and consequences of diabetes.	
Challenging maladaptive illness perceptions.	Increasing outcome expectancies for self-management and diabetes treatment by increasing perceptions of seriousness and controllability.	Providing medical information to change maladaptive illness perceptions, primarily focussing on the seriousness and controllability of the illness.	
**Session 2**			Empowerment
Exploring goals and developing personalized action plans	Increasing feelings of self-efficacy and empowerment through working on feasible and specific formulated goals for behavior change.	Sharing personal diabetes-related goals. Developing step-wised, specific action plans for these goals in subgroups.	
**Session 3**			Social support
Exploring and discussing (un)helpful ways of support.	Creating awareness on (un)helpful ways of support and the possible gap between wanted and received support.	Discussing ways of support that is perceived helpful and which ways of support are currently received.	
Developing personalized pro-active action plans	Overcoming barriers in behavior change by (asking) support from others.	Integrating help or support from others in the action plan.	
**Booster session**			
Reflections	Providing a reminder of the techniques learned during the course.	Sharing experiences on achieving the goals and action plans during the last three months. Questions or challenges that have arisen during the three months after the 3^rd^ course session are discussed.	

#### Control condition

Participants in the control group were invited to a single 2-hour educational lecture, in which they received information about living with diabetes from a medical perspective. A professor in general practice and diabetes care provided information on the course of diabetes (including treatment options and potential complications) and the latest developments in diabetes research, according to the classical didactical method.

### Measurements and measures

Questionnaires were administered at baseline, immediately after the intervention program (two months after baseline), and six months after the intervention (eight months after baseline). Participants in the control group also received a questionnaire at two months and at eight months after the baseline measurement.

#### Primary outcome measures

Self-care was assessed with the revised Summary of Diabetes Self-Care Activities measure (SDSCA) [34], assessing six separate self-care domains: exercise behaviors (2 items, Cronbach’s α = .75), glucose monitoring (2 items α = .50), foot care (2 items α = .68), general diet (2 items, α = .83), specific diet (2 items, α = .08), and smoking (1 item). With the exception of smoking, the self-care behaviors were rated on a 8-point scale, assessing the average number of days self-care was performed during the previous week (0–7 days). Because of the low internal reliability of the specific diet scale, the two items (fruit/vegetable intake and low-fat diet) were analyzed separately, as also suggested by Toobert et al. [34]. Smoking was assessed dichotomously (yes/no).

Diabetes-related distress was measured with the Problem Areas in Diabetes scale (PAID) [35]. The 20 items of the PAID assess experienced levels of diabetes-related emotional distress or problems on a 5-point Likert scale ranging from 0 (no problem) to 4 (a serious problem). The sum of the 20 items were transformed into an overall score between 0 and 100 (α = .95) by multiplying them by 1.25.

#### Proximal effect measures

Illness perceptions were measured with the revised Illness Perceptions Questionnaire (IPQ-R) [36]. In the first section of this questionnaire, participants indicate whether or not they experience 14 different symptoms (yes/no) and whether they attribute these to their diabetes (yes/no). The sum of the yes-rated items that were attributed to diabetes formed the ‘identity’ subscale (range: 0–14).

The second section of the IPQ-R (see S1 Box) assesses seven illness perception dimensions: ‘time-line acute/chronic’ (6 items, e.g. “My diabetes will last for a long time”,α = .86); ‘time-line cyclical’ (4 items, e.g. “My diabetes is very unpredictable”, α = .89); ‘consequences’ (6 items, e.g. “My diabetes is a serious condition”, α = .75); ‘personal control’ (6 items, “The course of my diabetes depends on me”, α = .72); ‘treatment control’ (5 items, “My treatment can control my diabetes”, α = .53); ‘coherence’ or understanding of diabetes (5 items, “My diabetes doesn’t make any sense to me”, α = .80) and ‘emotional representation’ (6 items, “When I think about my diabetes I get upset”, α = .83). The third section assesses causal beliefs (18 items) divided in three scales, based on factor analyses and as suggested by Moss-Morris et al [22]: own behavior in the past (6 items, e.g. “Diet or eating habits”, α = .83), psychological cause (5 items, e.g. “Stress or worries”, α = .72), and chance/bad luck (1 item). Apart from the ‘identity’ scale, which was measured dichotomously, the items of the IPQ-R were measured on a 5-point Likert scale ranging from 1 (strongly disagree) to 5 (strongly agree) and calculated into a mean score for each subscale.

Attitudes towards diabetes were measured with the Diabetes Attitude Scale (DAS-3) [37] (see S1 Box), consisting of five subscales: need for special training (5 items α = .73); perceived seriousness (7 items α = .73); value of tight control (7 items α = .56); psychosocial impact (6 items α = .66); and patient autonomy (8 items α = .65). The items are scored on a five-point Likert scale ranging from 1 (strongly disagree) to 5 (strongly agree) and calculated into a mean score for each subscale.

Perceptions of partner support were assessed by the questionnaire developed by Buunk et al. [38] (see S1 Box), measuring three dimensions of partner support: active engagement (5 items α = .91); protective buffering (8 items α = .73) and overprotection (6 items α = .74). The items were measured on a 5-point Likert scale ranging from 1 (never) to 5 (always) and calculated into a mean score for each subscale.

Empowerment was assessed by the Dutch Diabetes Empowerment Scale (Dutch DES-20) [39]; a Dutch version of Anderson’s Diabetes Empowerment Scale (DES) [40]. The Dutch-DES-20 consists of 20 items with a five-point Likert scale, ranging from 1 (totally disagree) to 5 (totally agree), and results in an overall empowerment mean score (α = .94).

#### Sociodemographic and clinical characteristics

Age, gender and diabetes duration were derived from patients’ health records kept by the GPs. All other sociodemographic and clinical characteristics were self-reported by the patients. Level of education was categorized into low (no education, primary school or low vocational training), middle (high school or middle vocational training) and high (college or university), based on the reported highest type of education completed. Marital status was dichotomized into ‘married or cohabiting’ and ‘other’ (single, divorced, widowed, other). Diabetes treatment was categorized into 1) lifestyle advice only, 2) oral hypoglycemic agents, and 3) insulin. To assess diabetes-related microvascular complications, patients were asked to indicate whether they suffered from 1) eye problems: retina problems (retinopathy), 2) kidney-problems: proteinuria or dialysis (nephropathy), 3) nerve damage (neuropathy), and 4) foot problems (wounds, amputation, need for adapted shoes). The presence of comorbidity was assessed by asking patients to indicate whether they suffered from 1) heart and vessel disease (e.g. serious heart condition or infarction), 2) cancer, 3) respiratory problems (asthma, chronic obstructive pulmonary disease (COPD), 4) joint conditions (neck and back problems, osteoporosis, arthrosis, rheumatoid arthritis) or 5) ‘other’

#### Statistical analysis

Participants’ characteristics are reported for the intervention group and control group separately. Differences in baseline characteristics between drop-outs and non-drop outs were tested with Student’s t-tests, and Chi-square or Fishers’ Exact tests. Multilevel analyses (MLA) were performed to test the effectiveness of the intervention over time, taking the dependency of the three measurements within participants into account. Analyses were performed according to the intention-to-treat-principle.

All outcome measures were analysed separately as dependent continuous variables in two level (patient and measurement) multivariate regression models. Condition (intervention or control group) and measurement (baseline, immediately after the intervention and after six months follow-up; dichotomous) and their interaction term (condition*measurement) were included in the analyses to examine the differences in effect between the groups over time. All analyses were adjusted for gender and age. All outcome measures were analyzed by linear regression models, except for smoking which was analyzed by a logistic regression model. Mixed effect models were used to calculate the intevention’s effect over time, with individual level being estimated as a random effect, and all other variables (age, sex and measurement*condition) as fixed effects.

Intervention effects were tested two-tailed and the significance level was set at p < .05. All analyses were performed with STATA 13.

## Results

**Fig 1:**
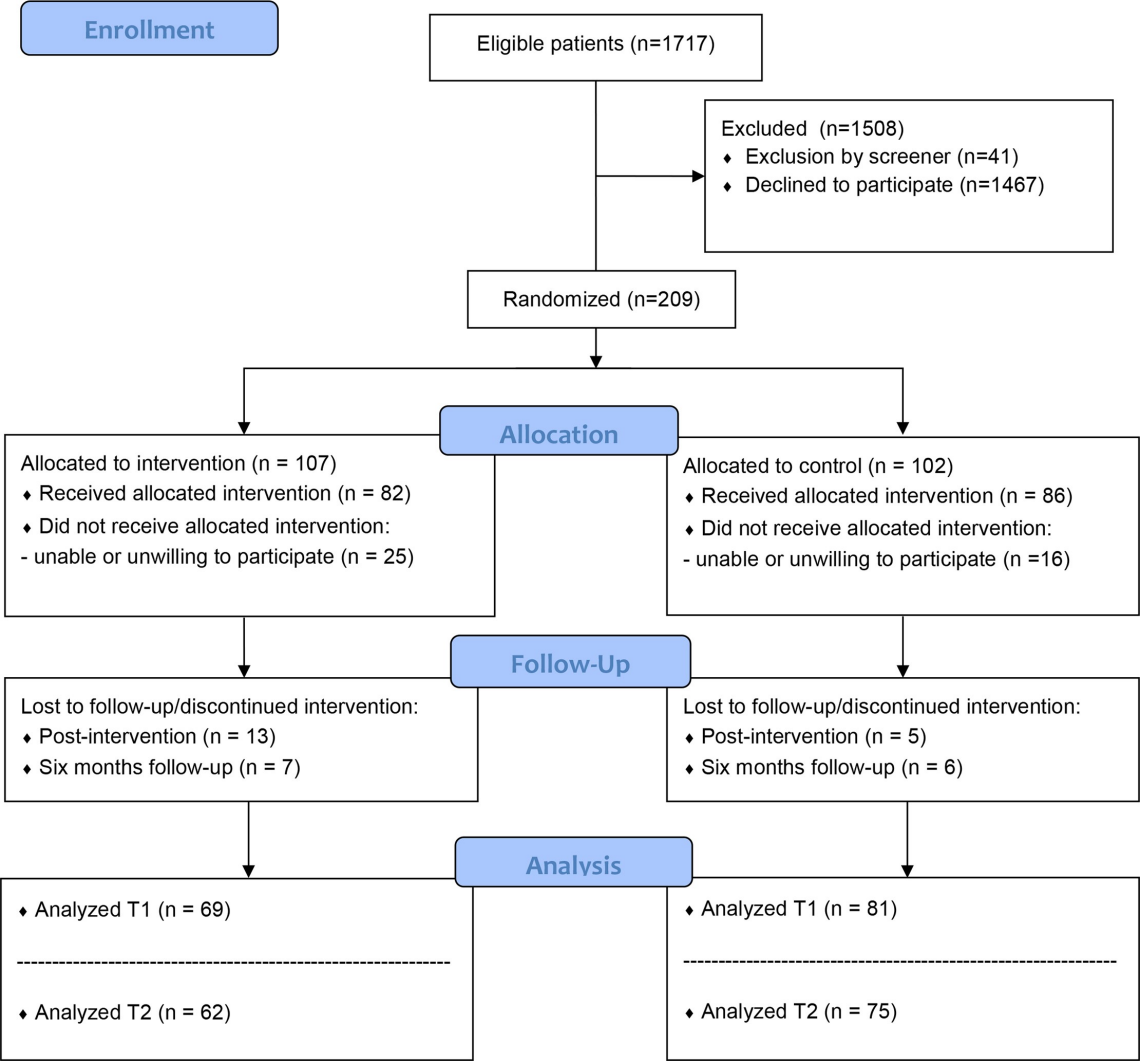
Flow-chart of participants.

Data were available from 168 participants in the study, with 82 participants in the intervention group and 86 participants in the control group (Fig 1). Even though the intervention and control group seem to somewhat differ in their sociodemographic and clinical characteristics (Table 2), these differences were not significant. Participants did not significantly differ from non-participants, with the exception of education level, which was higher among participants [41]. Thirty-one patients (18%) were lost during follow-up, with higher dropout rates in the intervention group (24%; n = 20) than in the control group (13%; n = 11). Non-response analyses showed that those lost to follow-up had a shorter diabetes duration (mean 2.1 vs. 2.5 years, p < 0.01) than those who completed the study. Furthermore, lower baseline scores on general dietary behaviors (mean 4.6 vs 5.3, p = 0.04), and psychological impact of type 2 diabetes (mean 2.8 vs. 3.1, p = 0.02) were found among dropouts, compared to participants with complete follow-up. In total, nine course groups were organized with an average of 8–9 participating patients per group. A little over half of the participants in the intervention group (54%) brought a partner to the course sessions.

**Table 2:** Baseline demographic and illness-related characteristics of the participants by study group; percentages or means (SD).

	Intervention (n = 82)	Control (n = 86)
Age (years), mean (SD)	63.5 (10.2)	63.7 (10.2)
Gender: male (%)	58.5 (n = 48)	52.3 (n = 45)
Education level (%)		
Low	27.9 (n = 22)	27.01 (n = 23)
Middle	43.0 (n = 34)	50.6 (n = 43)
High	29.1 (n = 23)	22.4 (n = 19)
Married or cohabiting (%)	79.8 (n = 63)	74.1 (n = 63)
Working population (%)	34.2 (n = 27)	24.7 (n = 21)
Diabetes duration (years), mean (SD)	2.4 (0.8)	2.4 (0.8)
Treatment (%)		
Lifestyle advice only	27.9 (n = 22)	37.7 (n = 32)
Oral hypoglycemics	68.4 (n = 54)	61.2 (n = 52)
Insulin	3.8 (n = 3)	1.2 (n = 1)
Complications: present (%)	19.5 (n = 15)	28.6 (n = 24)
Eye problems	6.5 (n = 5)	11.9 (n = 10)
Kidney problems	1.3 (n = 1)	2.4 (n = 2)
Nerve problems	1.3 (n = 1)	7.1 (n = 6)
Foot problems	10.4 (n = 8)	13.1 (n = 11)
Comorbid conditions: present (%)	61.5 (n = 48)	69.1 (n = 58)
Heart- and vessel condition	15.4 (n = 12)	23.8 (n = 20)
Cancer	1.3 (n = 1)	1.2 (n = 1)
Lung conditions	12.8 (n = 10)	17.9 (n = 15)
Joint conditions	33.3 (n = 26)	35.7 (n = 30)
Other conditions	15.4 (n = 12)	27.4 (n = 23)

### Effects on diabetes self-care and distress

Multilevel analyses (Table 3) showed that, immediately after the program, the intervention group showed a significantly higher increase in physical activity (mean estimated difference 0.76, p = .009) and fruit and vegetable intake (mean estimated difference 0.68, p = .014) than the control group. At the follow-up six months after the intervention, these effects had disappeared. Regarding diabetes-related distress, neither immediate nor six-month effects were found. Baseline levels of self-care appeared to be somewhat higher in the intervention group, but not significantly.

**Table 3:** Effects of a group-based intervention for people with type 2 diabetes on self-care behaviors and diabetes distress immediately post-intervention and six months after the intervention, adjusted for age and sex.

	Baseline		Immediately post-intervention			Six months post-intervention		
	*Unadjusted*		*Unadjusted*		*Adjusted*	*Unadjusted*		*Adjusted*
	Mean (SD)		Mean (SD)		Mean estimated difference (95% CI)	Mean (SD)		Mean estimated difference (95% CI)
	Intervention (n = 82)	Control (n = 86)	Intervention (n = 69)	Control (n = 81)		Intervention (n = 62)	Control (n = 75)	
*Self- care (0–7)*								
Exercise	4.5 (1.8)	4.3 (2.0)	4.9 (1.6)	4.0 (1.9)	0.76 (0.19–1.34)**	4.4 (1.7)	4.3 (2.0)	-0.04 (-0.64–0.55)
Glucose testing	0.5 (1.3)	0.3 (0.8)	0.7 (1.6)	0.4 (1.1)	0.14 (-0.28–0.56)	0.4 (1.3)	0.7 (1.6) †	-0.35 (-0.79–0.08)
Foot care	1.3 (2.0)	1.3 (1.8)	1.7 (2.1)	1.2 (1.8)	0.42 (-0.18–1.02)	1.5 (1.8)	1.4 (2.0)	0.06 (-0.56–0.68)
Diet: general	5.3 (1.4)	5.1 (1.8)	5.4 (1.3)	4.9 (1.7)	0.29 (-0.18–0.76)	5.3 (1.2)	4.8 (1.8)	0.25 (-0.24–0.74)
Diet: fruit/vegetables	5.2 (1.9)	5.4 (1.9)	5.7 (1.5) †	5.3 (1.7)	0.68 (0.14–1.22)*	5.3 (1.6)	5.6 (1.6)	-0.16 (-0.72–0.39)
Diet: low fat	5.0 (1.9)	4.6 (2.1)	4.7 (2.0)	4.8 (1.9)	-0.48 (-1.14–0.18)	4.5 (2.2) †	4.2 (2.1)	-0.09 (-0.77–0.59)
Non-smoking (%)	87.2	77.1	87.0	74.4	0.85 (-3.36–5.06)	88.7	81.4	-3.74 (-8.09–0.61)
Diabetes distress (0–100)	12.6 (14.3)	13.9 (16.0)	15.4 (14.7)	14.9 (15.7)	1.80 (-.157–5.17)	13.5 (13.3)	12.7 (15.1)	1.57 (-1.92–5.06)

† Significant difference from baseline at the 0.05 level

* Significant intervention effect at the 0.05 level.

** Significant intervention effect at the 0.01 level

### Effects on illness perceptions, attitude, empowerment and partner support

Multilevel analyses (Table 4) showed that, immediately after the program, the intervention group showed a significantly higher belief in diabetes being caused by chance/bad luck (mean estimated difference 0.45, p = .039 and higher feelings of empowerment (mean estimated difference 0.21, p = .006) than the control group. Six months after the intervention, the differences in empowerment were still present (mean estimated difference 0.16, p = .044). The control group, on the other hand, showed a significantly higher increase in perceiving type 2 diabetes as having a chronic timeline (mean estimated difference 0.22, p = .029) at the six-month follow-up than the intervention group. No significant effects were found on partner support; neither immediately after the intervention nor six months after the intervention.

**Table 4:** Effects of a group-based intervention for people with type 2 diabetes on cognitions, empowerment and partner support, immediately post-intervention and six months after the intervention, adjusted for age and sex.

	Baseline		Immediately post-intervention			Six months post-intervention		
	*Unadjusted*		*Unadjusted*		*Adjusted*	*Unadjusted*		*Adjusted*
	Mean (SD)		Mean (SD)		Mean estimated difference (95% CI)	Mean (SD)		Mean estimated difference (95% CI)
	Intervention (n = 82)	Control (n = 86)	Intervention (n = 69)	Control (n = 81)		Intervention (n = 62)	Control (n = 75)	
*Illness perceptions (1–5)*								
Identity (0–14)	0.69 (1.41)	0.72 (1.49)	0.65 (1.50)	0.69 (1.27)	0.02 (-0.38–0.42)	0.53 (1.24)	0.50 (1.20)	0.06 (-0.35–0.47)
Timeline: chronic	3.92 (0.68)	3.83 (0.79)	4.08 (0.72) †	3.99 (0.67)	0.02 (-0.17–0.21)	3.87 (0.69)	4.02 (0.74) †	-0.22 (-0.41 –-0.02)*
Timeline: cyclical	2.35 (0.85)	2.42 (0.77)	2.43 (0.79)	2.45 (0.74)	0.03 (-0.22–0.28)	2.48 (0.90)	2.43 (0.80)	0.12 (-0.14–0.37)
Consequences	2.53 (0.66)	2.54 (0.68)	2.81 (0.60) †	2.65 (0.61)	0.17 (-0.01–0.35)	2.76 (0.57) †	2.60 (0.57)	0.18 (0.00–0.36)
Control: personal	3.88 (0.58)	3.69 (0.55)	4.02 (0.51)	3.66 (0.56)	0.10 (-0.08–0.28)	3.94 (0.44)	3.78 (0.56)	-0.08 (-0.27–0.10)
Control: treatment	3.81 (0.46)	3.71 (0.51)	3.90 (0.50)	3.70 (0.47)	0.08 (-0.08–0.24)	3.79 (0.45)	3.73 (0.55)	-0.04 (-0.20–0.13)
Illness coherence	3.50 (0.68)	3.29 (0.82)	3.71 (0.76) †	3.43 (0.77)	0.07 (-0.13–0.28)	3.64 (0.70)	3.45 (0.65)	0.02 (-0.19–0.24)
Emotional representations	2.21 (0.63)	2.22 (0.68)	2.20 (0.63)	2.26 (0.67)	-0.05 (-0.24–0.14)	2.18 (0.66)	2.28 (0.62)	-0.10 (-0.30–0.09)
Cause: psychological	2.19 (0.81)	2.23 (0.75)	2.32 (0.79)	2.40 (0.84) †	-0.04 (-0.23–0.15)	2.31 (0.81)	2.27 (0.74)	0.03 (-0.17–0.22)
Cause: risk behavior	2.51 (0.77)	2.43 (0.69)	2.56 (0.80)	2.52 (0.66)	-0.10 (-0.29–0.10)	2.50 (0.82)	2.50 (0.73)	-0.16 (-0.36–0.04)
Cause: bad luck	2.53 (1.22)	2.77 (1.15)	2.91 (1.14) †	2.67 (1.15)	0.45 (0.03–0.87)*	2.75 (1.09)	2.90 (1.07)	0.06 (-0.37–0.50)
*Attitude (1–5)*								
Need for special training	4.04 (0.54)	4.01 (0.54)	4.10 (0.57)	4.07 (0.60)	0.00 (-0.18–0.19)	4.09 (0.49)	4.06 (0.57)	0.01 (-0.18–0.20)
Seriousness	3.36 (0.61)	3.34 (0.53)	3.64 (0.58) †	3.45 (0.51)	0.15 (0.00–0.29)	3.57 (0.56) †	3.46 (0.52)	0.06 (-0.09–0.22)
Value of tight control	3.75 (0.40)	3.59 (0.41)	3.85 (0.48)	3.62 (0.47)	0.06 (-0.09–0.20)	3.73 (0.46)	3.57 (0.44)	0.00 (-0.15–0.14)
Psychological impact	3.03 (0.55)	3.12 (0.54)	3.15 (0.49)	3.05 (0.63)	0.15 (0.00–0.31)	3.15 (0.50)	3.12 (0.54)	0.09 (-0.07–0.25)
Autonomy	3.24 (0.42)	3.20 (0.55)	3.29 (0.46)	3.21 (0.46)	0.02 (-0.13–0.17)	3.32 (0.49)	3.22 (0.54)	0.04 (-0.11–0.20)
Empowerment (1–5)	3.65 (0.50)	3.60 (0.53)	3.89 (0.51) †	3.64 (0.50)	0.21 (0.06–0.37)**	3.86 (0.44) †	3.67 (0.46)	0.16 (0.00–0.32)*
*Partner support (1–5)*								
Active engagement	3.38 (0.83)	3.07 (1.00)	3.48 (0.76)	3.04 (0.84)	0.16 (-0.05–0.37)	3.42 (0.80)	3.01 (0.84)	0.00 (-0.21–0.21)
Protective buffering	2.22 (0.71)	2.27 (0.63)	2.22 (0.64)	2.31 (0.66)	-0.03 (-0.23–0.18)	2.20 (0.63)	2.29 (0.72)	-0.02 (-0.23–0.19)
Overprotection	1.91 (0.63)	1.75 (0.59)	1.99 (0.60)	1.71 (0.55)	0.07 (-0.11–0.25)	2.02 (0.73)	1.73 (0.59)	0.05 (-0.14–0.23)

† Significant difference from baseline at the 0.05 level

* Significant intervention effect at the 0.05 level.

** Significant intervention effect at the 0.01 level

## Discussion

This study examined the immediate and six-month effectiveness of a group-based self-management support program for people with type 2 diabetes one to three years after diagnosis, and their partners. The intervention group showed more positive results regarding diabetes self-care, and empowerment than the control group. At six-months follow-up, the behavioral differences between the two groups had disappeared, while the differences in empowerment had persisted. Neither immediate nor six-month effects of the program were found on patients’ levels of diabetes-related distress. Apparently, sustainable changes in patients’ empowerment do not necessarily lead to sustainable changes in their behavior.

These results contribute to the still relatively sparse knowledge on the effectiveness of support programs that aim to improve diabetes self-care by targeting diabetes-related perceptions. We based our program on the assumption that more adaptive illness perceptions–in particular those concerning the seriousness of type 2 diabetes and its controllability by medical treatment and own behavior–would positively influence participants’ diabetes self-care and distress. These theoretical assumptions seem to be only partly supported by the short-term intervention effects found in this study. Immediately after completion of the intervention program, participants reported higher levels of physical activity and fruit and vegetable intake than participants in the control group. In addition, participants in the intervention group felt more empowered to make treatment decisions. No (significant) changes, however, were found in the participants’ cognitions regarding the seriousness and controllability of their condition, even though these were hypothesized to be important preconditions for behavioral change; particularly in this patient group. Furthermore, six months after the intervention program, the increased physical activity and fruit and vegetable intake of the participants in the intervention program had diminished, whereas the differences in empowerment between the intervention and control group had remained. Results from the two previous CSM-based studies in diabetes [9,26] showed comparable modest or short-term intervention effects on self-care, although they did find sustained changes in illness- and treatment-related perceptions in their studies. Hence, these findings suggest that even if CSM-based self-management support effectively alters patients’ cognitions, and as such establishes the necessary psychological conditions for behavioral change, this still appears to be insufficient to maintain healthy behaviors on the longer term. Achieving sustainable improvements in physical activity and diet are, however, considered to be among the most challenging health behaviors [6,42,43] and therefore require more intensive and prolonged support. Also, diabetes-related distress remained unaffected by the intervention over time. Levels of distress among participants were, however, already very low at baseline, which left little room for improvement by the program. Absence of diabetes-related distress during the first years after diagnosis might rather reflect the presence of misconceptions about the seriousness of type 2 diabetes and not being fully engaged in self-care than being an indication of successful adaptation to living with the condition [6]. The changes in lifestyle did not result in heightened distress levels, which might suggest that the intervention provided reassurance and helped participants to identify successful and acceptable methods to perform diabetes self-care. Hence, the mere finding that distress levels remained stable over time, might be an indication of the effectiveness of the intervention in itself. Nonetheless, we cannot rule out that the intervention did not work as intended.

There are some study limitations that need to be addressed. First, participants in our study may not have been fully representative for the target population, which could have implications for the generalizability of the study results. The low eventual participation rate of 10%, and rather high and non-random drop-out rate of 18% during the study seem to indicate that the study results especially apply to people with type 2 diabetes who are motivated to self-manage their condition. Also, participants appeared to be higher educated than patients who declined the invitation to participate, which could indicate that our study was less able to reach those patient populations that are generally more prone to unfavorable (health) outcomes [44–47]. Furthermore, it should be noted that half of the participating patients did not bring a partner to the course sessions which, considering the known important role of partners in diabetes management [19–21], could have had its implications on the effectiveness of the intervention. Unfortunately, our study sample does not allow for subgroup analyses to test for differences in results between patients with and patient without a partner. Furthermore, the control condition was not equivalent to the intervention condition regarding the frequency and duration of the sessions. Nevertheless, participants in the control group did receive an educational lecture, and as such received at least some kind of intervention (attention) as well. Another potential limitation is that all study outcomes were self-reported, and no clinical outcomes were included as more objective determinants of diabetes self-management. And even though this study was primarily focused on diabetes outcomes as perceived by the participants, the assessed self-care behaviors–one of the primary outcome measures–may have been subjected to social desirability bias [48]. Finally, many variables were tested in this study, for we wanted to capture the broad sense of self-care and gain more insight into the changes in its underlying factors. As a consequence of this multiple testing strategy, it should be kept in mind that some of the statistically significant effects could have occurred by chance.

In conclusion, our group-based self-care support intervention for people with type 2 diabetes appeared to have had short-term favourable effects on patients’ lifestyles and feelings of empowerment. However, in order to achieve sustainable behavioral changes, more prolonged support is necessary. For it does not appear feasible to motivate the often asymptomatic T2DM patient population to engage in more course sessions or educational training in addition to regular diabetes consultations during their first phases of illness, we believe that aspects from this intervention should rather be integrated within regular care and be monitored, for instance, in yearly comprehensive assessments and the resulting individual care plan. Regular and repeated discussion of patients’ and partners’ perceptions could help health care providers in identifying maladaptive beliefs that negatively affect behavioral and emotional management of diabetes, while the development of realistic action plans and regular discussion of its progress and the obstacles encountered may help patients stay focused on working towards their long-term goals.

## Supporting information

S1 Checklist CONSORT 2010 checklist. (DOC)

S1 Table Three-item screener. (DOCX)

S1 Box Description of the scores on the subscales of the IPQ-r, DAS-3, and the partner support questionnaire. (DOCX)

S1 Data File (ZIP)
