# Supplementary material for: Effectiveness of a self-management support program for type 2 diabetes patients in the first years of illness: Results from a randomized controlled trial
Source: PLoS One. 2019 Jun 27;14(6):e0218242. doi: 10.1371/journal.pone.0218242 (PMC6597059; doi:10.1371/journal.pone.0218242)
Supplement: S1 Table — (DOCX) [file pone.0218242.s002.docx]

**S1 Table. Three-item screener**

| **1. How much uncertainty do you currently experience in your life as a result of being diabetic?** | | | | |
| --- | --- | --- | --- | --- |
| none at all  (0) | slight amount  (1) | moderate amount  (1) | large amount  (1) | extremely large amount  (1) |
| **2. How effective are you in coping with your diabetes?** | | | | |
| not at all  (1) | slightly effective  (1) | moderately effective  (1) | very effective  (0) | extremely effective  (0) |
| **3. My diabetes has major consequences on my life.** | | | | |
| strongly disagree  (0) | disagree  (1) | neither agree nor disagree  (1) | agree  (1) | strongly agree  (1) |
