# Supplementary material for: Effectiveness of a self-management support program for type 2 diabetes patients in the first years of illness: Results from a randomized controlled trial
Source: PLoS One. 2019 Jun 27;14(6):e0218242. doi: 10.1371/journal.pone.0218242 (PMC6597059; doi:10.1371/journal.pone.0218242)
Supplement: S1 Box — (DOCX) [file pone.0218242.s003.docx]

**S1 Box: Description of the scores on the subscales of the IPQ-r, DAS-3, and the partner support questionnaire.**

| **IPQ-r** | |
| --- | --- |
| **Subscale** | **Description** |
|  |  |
| Identity | Higher scores indicate the presence of more symptoms, which are being attributed to diabetes by the individual. |
| Timeline acute/chronic | Higher scores indicate higher beliefs of diabetes being a long-term (or life-long) condition. |
| Timeline cyclical | Higher scores indicate higher beliefs of diabetes being a condition that changes over time. |
| Consequences | Higher scores indicate higher beliefs of diabetes being a (potentially) serious condition with a higher perceived impact on the individual’s own life, and that of their close associates. |
| Personal control | Higher scores indicate higher beliefs in the individual’s personal effectiveness and ability to control their diabetes. |
| Treatment control | Higher scores indicate higher beliefs regarding the effectiveness of (medical) treatment, and the effectiveness of health care providers in diabetes control. |
| Illness coherence | Higher scores indicate a greater perceived understanding of diabetes, with diabetes making ‘more sense’ to the individual. |
| Emotional representations | Higher scores indicate more emotional feelings towards living with diabetes, e.g. anger or fear. |
| Cause: own behavior | Higher scores indicate higher beliefs of own behaviors (e.g. diet) being a cause for diabetes. |
| Cause: psychological cause | Higher scores indicate higher beliefs of psychological factors (e.g. stress) being a cause for diabetes. |
| Cause: chance/bad luck | Higher scores indicate higher beliefs of chance or bad luck being a cause for diabetes. |
| **DAS-3** | |
| **Subscale** | **Description** |
|  |  |
| Need for special training | Higher scores indicate a higher perceived need for health care professionals to have had special training in teaching, counselling and techniques for behaviour change. |
| Seriousness of T2DM | Higher scores indicate higher beliefs of diabetes being a serious condition. |
| Value of tight control | Higher scores indicate higher beliefs of the benefits of tight glucose control outweighing its ‘costs’ in patients. |
| Psychosocial impact | Higher scores indicate higher beliefs of diabetes having a psychosocial impact on patients’ lives. |
| Patient autonomy | Higher scores indicate higher beliefs of patients being the primary decision-maker regarding their diabetes and its treatment. |
| **Partner support questionnaire** | |
| **Subscale** | **Description** |
|  |  |
| Active engagement | Higher scores indicate more active engagement and support in helping partners use constructive problem-solving skills to manage their diabetes. |
| Protective buffering | Higher scores indicate more effort in hiding their own concerns and emotions regarding their partners’ diabetes in order to protect them. |
| Overprotection | Higher scores indicate more (excessive) efforts in protecting their partners from the challenges of diabetes management. |
